# Food Security and Diet Quality in Native Hawaiian, Pacific Islander, and Filipino Infants 3 to 12 Months of Age

**DOI:** 10.3390/nu12072120

**Published:** 2020-07-17

**Authors:** Sally Campbell, John J. Chen, Carol J. Boushey, Heather Eicher-Miller, Fengqing Zhu, Marie K. Fialkowski

**Affiliations:** 1 Technological University Dublin, Kevin Street, Saint Peter’s, D08X622 Dublin 2, Ireland; campbes7@tcd.ie; 2 Trinity College University of Dublin, College Green, Dublin 2, Ireland; 3 Department of Human Nutrition, Food and Animal Sciences, College of Tropical Agriculture and Human Resources—University of Hawai‘i at Mānoa, Honolulu, HI 96822, USA; jjchen@hawaii.edu; 4 Epidemiology Program, University of Hawaii Cancer Center, 701 Ilalo St, Honolulu, HI 96813, USA; cjboushey@cc.hawaii.edu; 5 Department Nutrition Science, College of Health and Human Science, Purdue University, West Lafayette, IN 47907, USA; heicherm@purdue.edu (H.E.-M.); zhu0@purdue.edu (F.Z.)

**Keywords:** infants, minority, food security, diet diversity, diet quality

## Abstract

Food insecurity and other nutritional risks in infancy pose a lifelong risk to wellbeing; however, their effect on diet quality in Native Hawaiian, Pacific Islander, and Filipino (NHPIF) infants in Hawai‘i is unknown. In this cross-sectional analysis, the association between various indicators of food security and NHPIF infant diet quality were investigated in 70 NHPIF infants aged 3–12 months residing on O‘ahu, Hawai‘i. The dietary assessments of the infants were collected using a mobile food record^TM^. Foods consumed across four days were categorized into seven food groups. Indicators for food security were examined through an adapted infant food security index and other indicators. Data were analyzed using chi-square tests, independent sample t-tests, multinomial logistic regression, and linear regression models. In models adjusting for age and sex, infants defined as food insecure by the adapted index were found to consume foods from more food groups and consume flesh foods on a greater proportion of days. Of the indicators examined, the adapted index was shown to be the best indicator for food group consumption. Further work is needed on a more representative sample of NHPIF infants to determine the impact that food security has on nutritional status and other indicators of health.

## 1. Introduction

Food insecurity is defined as limited or uncertain availability of nutritionally adequate foods. It is considered a high priority for public health stakeholders given its economic and health impacts and the associated nutritional risks [1]. These impacts include worse developmental outcomes and chronic illness among children [2], and poorer health outcomes in infants [3]. Situations of food insecurity are linked with disrupted eating patterns, poor diet quality and nutritional inadequacy across age groups and demographics [4]. Infants aged 0–12 months are more susceptible to the adverse effects of food insecurity given their high nutritional requirements for growth and dependence on others for nutrition [3]. Optimal nutrition during infancy protects against morbidity and mortality, reduces the risk of chronic disease, and promotes better overall development, and thus efforts to understand and mitigate nutritional risks such as infant food insecurity and improved nutrition in early life may have far-reaching implications [5].

An eighteen-item survey known as the U.S. Household Food Security Survey Module (USHFSSM) was developed by the United States Department of Agriculture (USDA) to assess household food security; a portion of the survey questions may be used to determine the food security of a child or children within the household [6]. In contrast to this method, Schlichting D. et al. devised a food security index which aims to assess the food security of an infant at an individual level within a household [3]. In addition to food security status, another indicator of nutritional risk is household income as it is theorized that some low-income households lack economic access to healthy foods [7,8]. Similarly, eligibility to food assistance programs, such as the Special Supplemental Nutrition Program for Women, Infants and Children (WIC) and the Supplemental Nutrition Assistance Program (SNAP), are based on household income criteria and may also indicate nutritional risk [8,9,10]. The separate associations between each of these indicators of nutritional risk: food insecurity, household income, and food assistance program participation, with overall dietary quality, reveals disagreement across the literature. These nutritional risks may have contrasting influences on the diet quality of different demographics, namely by age and ethnic group.

Food insecurity disproportionately influences households headed by individuals of minority race/ethnicity [4]. For example, the odds of food insecurity were higher among ethnic minority infants including Maori, Pacific Islander, and Asian infants when compared to all other infants, in a representative sample of the New Zealand infant population [3]. This race/ethnicity disparity in food insecurity extends to associations with poor dietary intake among food-insecure minority groups. Leung C et al. studied a population of 4393 adults from the National Nutrition and Health Examination Survey (NHANES) and found food insecurity was associated with a lower diet quality indicated by Healthy Eating Index (HEI) score, and this association was most pronounced among those who identify as American Indian or Alaska Native, Native Hawaiian or Pacific Islander or as multiracial [4]. Despite their inclusion in this sample, the Native Hawaiian or Pacific Islander population represents a unique group within the US at risk of poorer health outcomes than the overall population [11]. Heinrich K. and colleagues found that a combination of high living costs and low-income negatively impacts some low-income residents in Hawai‘i, contributing to food insecurity [12].

Minority groups such as Native Hawaiian, Pacific Islander or Filipinos (NHPIF) in Hawai‘i report higher levels of food insecurity than other ethnicities [13]. Yet the relationship between food insecurity and other indicators of nutritional risk, household income and food assistance participation, to diet quality among NHPIF infants in Hawai‘i is not known, nor is which indicator has a stronger relationship to dietary quality. Therefore, the objective of this study was to determine which indicator of nutritional risk would have the strongest association with the diet quality of NHPIF infants 3–12 months of age. This was assessed using responses to two questions modified from the USHFSSM relating to money running out for food and utilities, participation in food assistance programs, annual household income, or an adapted infant food security index. A Minimum Dietary Diversity (MDD) score is used when evaluating the diet quality of infants aged 6–12 months, and thus MDD was used for this analysis. The diet quality of infants aged 3–12 months was examined by food group consumption. Authors hypothesized that an adapted infant food security index, which takes into account multiple indicators of food insecurity, would have the strongest association with diet quality assessed using the MDD and food group consumption [14].

## 2. Materials and Methods

### 2.1. Study Sample

The target population for this cross-sectional study was NHPIF infants between 3–12 months of age residing on O‘ahu, Hawai‘i. To be eligible to participate in the study, the infant’s caregiver(s) had to be 18 years of age or older, have an iOS mobile device, and have reliable access to the Internet. The infant participants had to have commenced complementary feeding prior to study onset and be reported by the caregiver as at least part Native Hawaiian, Pacific Islander or Filipino. A convenient sample of NHPIF infants was primarily recruited through community-based events (e.g., Baby Expo), programs (e.g., WIC), and networking. Seventy infants and their caregivers completed the study, of which 56 of the infants were aged 6–12 months. Institutional Review Board (IRB) exemption from the University of Hawai‘i was received prior to the collection of data (IRB reference number: 2017-00845). Consent was obtained in writing from the caregivers for both their participation and their infant’s participation prior to collecting any data. Data was collected between March 2018–February 2019.

### 2.2. Participant Characteristics

At study onset, caregivers completed a questionnaire using a secure on-line web application. Topics included feeding practices followed prior to enrolment in the study. Demographic information included annual household income, information relating to household food security status including household participation in food assistance programs such as WIC, SNAP, free or reduced cost school meals, food banks since the child was born and two questions informed from the USHFSSM [6]. The two questions modified from the USHFSSM were:

1. In the past 12 months, how often did your money for food run out before the end of the month? (Never, Seldom, Sometimes, Most times, Always, Don not know, No response)
2. In the past 12 months, how often did your money for household utilities (e.g., water, fuel, oil, electricity) run out before the end of the month? (Never, Seldom, Sometimes, Most times, Always, Do not know, No response)

### 2.3. Dietary Assessment

Infant dietary assessment was completed through surrogate reporting via the caregiver with the mobile food record^TM^ (mFR^TM^) [15]. The mFR^TM^ is an application designed specifically for the assessment of dietary intake from the Technology Assisted Dietary Assessment^TM^ project (http://tadaproject.org/) which uses the camera on a mobile device to capture food and beverage intake, which is then used to estimate energy, nutrients, food and beverage intakes [15,16,17,18]. The mFR^TM^ was loaded on to the caregiver’s mobile device and training on the mFR^TM^ application was completed prior to data collection. Caregivers were instructed to take before and after images of all foods and beverages the participant consumed over a 4-day collection period (Thursday–Sunday). After the collection period concluded, a member of the research team reviewed the images with caregivers to verify content, as needed, and to probe for any forgotten foods or beverages. At the end of the data collection period, caregivers were compensated with a $40 gift card.

### 2.4. Dietary Diversity Score

The global metric Minimum Dietary Diversity (MDD) score from the World Health Organisation’s (WHO) indicators for assessing infant and young child feeding practices (IYCF, 2007) [14] was used to examine infant diet quality. Consuming a wide range of foods to meet one’s nutrient needs is one tenet of a healthy diet and, in infancy, the number of food groups consumed can predict the nutrient density of the diet [19]. Given the absence of an HEI for children aged below 2 years [20] and the limited selection of infant diet quality scoring metrics in the US, MDD was implemented as an indicator of the micronutrient adequacy of NHPIF infants. The WHO recommends the initiation of complementary feeding from 6 months onward [21]; thus, this assessment is appropriate only for infants aged 6 months and older. Solid foods and liquids consumed in any amount more than a condiment were enumerated over the 4 days using the mFR^TM^ images. Using the MDD metric, solids and liquids consumed in a day were categorized into seven food groups: (1) grains, roots, and tubers; (2) legumes and nuts; (3) dairy products (milk, including formula, yogurt, cheese); (4) flesh foods (meat, fish, poultry, liver/organ meats); (5) eggs; (6) vitamin A-rich fruits and vegetables; and (7) other fruits and vegetables. Particular attention is given by the WHO to assess vitamin A intake in children aged 6–59 months. This is because vitamin A deficiency in infancy is a public health problem in many developing countries. Furthermore, vitamin A deficiency can cause visual impairment and may increase the risk of illness and death from childhood infections [22]. MDD was considered met if the infant consumed four or more of the seven food groups, on average, each day and unmet if less than four food groups had been consumed, on average, each day. Human milk is not counted in a food group in the version of the WHO MDD metric used [14].

### 2.5. Adapted Food Security Index

A food security index was adapted from an index developed by Schlichting D. et al. and is outlined in Table 1 [3]. The adapted index estimates the degree of infant food security as a weighted sum of scores from two of the modified USHFSSM questions, i.e., use of defined methods to cope with food insecurity such as using food banks, and infant breastfeeding status at 3 months. Breastfeeding status at this age was chosen as each participant had commenced complementary feeding prior to study onset and the minimum participant age was 3 months. Positive points were awarded for breastfeeding to 3 months and never running out of money for food or utilities, while scoring was reversed and points deducted for the use of coping methods such as using food assistance programs. The range of scores was −14 to 4. For ease of discussion, a constant equal to the lowest value (−14) was added to all scores, shifting the range upward to 0–18 where 0 represents the lowest status of food security and 18 the highest. A cutoff for infant food insecurity was set at half a standard deviation below the mean (12.76). This cutoff point is consistent with other authors who claim it represents the minimum socially acceptable level of food insecurity prevalence. Infants were classified by this index into either extremely, highly or moderately food insecure or extremely, highly or moderately food secure (see Table 1 and Table 5) [3].

### 2.6. Analysis

Descriptive statistics were used to categorize the sample. Mean and standard deviations (SD) were used to describe age and food group consumption. Frequencies and percentages were used to describe food security prevalence by the various indicators. Participation in food assistance programs was examined from the dichotomous responses: yes or no. Responses to the modified USHFSSM questions on running out of money for food or utilities were collapsed into dichotomous variables (yes or no). The responses never or seldom to either question was reported as not experiencing (no) while the responses sometimes, most times or always were reported as experiencing it (yes). The mean daily consumption of the 7 food groups was calculated as a mean of all four mFR^TM^ days, a method commonly used [3,23]. The frequency of consumption of each food group with the average number of food groups consumed was examined in all participants (i.e., 3–12 months). Quantitative variables were compared between food-secure and food-insecure subgroups using both independent samples t-test methods and the non-parametric Mann–Whitney U tests. Independent sample t-test *p*-value results were presented when the results from the two approaches were similar. Categorical variables were compared using Chi-squared tests or Fisher’s exact tests [23]. Spearman’s rho correlation coefficient was used in examining correlation between food security indicators and food groups consumed [24]. The proportion meeting/not meeting the MDD (≥4 food groups) was determined in the participants 6–12 months of age subgroup only, across all four days of the mFR^TM^. A sequence of multivariable logistic regression and linear regression models were developed to investigate the relationships between MDD and individual and total food group intake, and those variables that yielded significant results in the bivariate analysis. In logistic regression analysis, MDD was met or not, on average, across the four days (yes or no) was used as the dependent variable to investigate its associations with the adapted food security index score, household income or the response to running out of money for food, adjusting for age and sex. In the linear regression analysis, total and individual food group consumption was used as the dependent variable to study its relationships with the adapted food security index score, household income, responses to running out of money for food and utilities or WIC and SNAP participation status, adjusting for age and sex as considered in similar studies [23]. Statistical significance was set at *p*-value < 0.05. All statistical analyses were performed using SPSS version 26 (Armonk, NY, USA). As this was a secondary analysis, a power calculation was not conducted to identify the sample size.

## 3. Results

### 3.1. Food Security Classification Using the Adapted Food Security Index

Of the 70 infants, approximately one quarter were classified as food insecure, with over 20% being classified as moderately or highly or extremely food insecure. Over 40% were highly food secure, while no infant was classified as extremely food secure (see Table 2).

### 3.2. Other Characteristics of Participants

Approximately half of the infants were girls and the mean age was 7.4 months. A greater proportion of food-secure infant caregivers were married and earning higher incomes. Likewise, a greater proportion of food-secure infant caregivers attended college (see Table 3).

Table 4 displays the proportion of infants classified as food secure or insecure by component on the food security index adapted from Schlichting D. et al. [3]. Most infants classified as food insecure were part of households who experienced running out of money for food or utilities by the end of the month. Additionally, over 80% of food-insecure infants’ households participated in WIC and over 50% participated in SNAP.

### 3.3. Average Percentage Daily Food Group Consumption by Food Security Status

The seven food groups used to classify the infants’ dietary characteristics are shown in Table 5. Grains, roots or tubers were the most commonly consumed foods followed by other fruits and vegetables and dairy products. The mean total number of food groups consumed daily was a little over 3.0 food groups with a range between 1.0–5.3. The mean consumption of food groups by infants, 3–12 months, from households who experience running out of money for food or utilities at the end of the month was almost four food groups each day, while infants from households who do not experience running out of money for food or utilities at the end of the month had, on average, three food groups each day. Infants, 3–12 months, from households not experiencing running out of money for food or utilities at the end of the month consumed a flesh food more than half of the time, while those who did not experience running out of money for food or utilities by the end of the month consumed a flesh food approximately 20% of the time. A marginal but statistically significant difference in the consumption of grains, roots and tubers, between those who do and do not experience running out of money for food, was also identified. Infants, 3–12 months, from households who do experience running out of money for food at the end of the month consumed a grain root or tuber almost 100% of the time, while those who do not experience running out of money for food by the end of the month consumed a grain, root or tuber approximately 80% of the time.

Only one marginal, but statistically significant, difference in infant daily food group consumption was identified between households who do and do not participate in WIC and SNAP. Infants in households who participate in WIC or SNAP consumed a grain, root or tuber over 90% of the time, while those who do not participate in WIC or SNAP consumed a grain, root or tuber approximately 80% of the time. On average, infants in households who participate in WIC or SNAP consumed foods from approximately 3.5 food groups each day, while infants in households who do not participate in WIC or SNAP consumed foods from approximately 3.0 food groups each day (see Table 5).

There was a significant difference in the intake of flesh foods between those infants who were and were not defined as food insecure by the adapted food security index. Those defined as food insecure had a flesh food intake over 60% of the time, while those defined as food secure had a flesh food intake more than 15% of the time. Likewise, infants defined as food insecure had a grain, root or tuber group over 95% of the time versus approximately 80% of the time by those defined as food secure. Furthermore, infants defined as food insecure had, on average, 3.76 out of seven food groups a day, while those defined as food secure had, on average, almost 3.0 food groups (see Table 5).

Weak to moderate, but statistically significant negative spearman correlations were observed between infant food security by the adapted index and total food group, the grain, root, and tuber food group and the flesh food group consumption. Likewise, weak, but significant negative spearman correlations were found between household income and total food group consumption (see Table 5).

### 3.4. Proportion of Infants Aged 6–12 Months Meeting the MDD by Food Security Indicators

Table 6 presents the proportion of infants 6–12 months who did and did not meet the MDD by the various indicators of food security examined in this study. The highest proportion of infants to meet the MDD were those classified as moderately food insecure by the adapted infant food security index, of whom over 70% met the MDD. Significantly more food-insecure infants met the MDD in comparison to food-secure infants. In addition, over two-thirds of infants from households with an annual income of <$35,000, met the MDD whereas a little less than 30% of infants from households with an annual income of >$35,000 met the MDD. Similarly, the income bracket with the highest proportion of infants (approximately 80%) meeting the MDD was <$10,000 while the lowest proportion of infants (over 15%) meeting the MDD were from the $60,000–75,000 bracket followed by the >$75,000 bracket with over 25%.

### 3.5. Infant Food Security Indicators and Food Group Consumption Examined Using Linear Regression Analysis

Presented in Table 7 are the statistically significant results of linear regression analysis between food security indicators and total food group consumption, grain, root, and tuber food group consumption, and flesh group consumption. The regression findings indicate a statistically significant association between food security score and grain, root, and tuber consumption. For each unit increase in food security classification from extremely food insecure to extremely food secure by the adapted food security index, the frequency of daily grain, root, and tuber consumption decreases by approximately 6% after controlling for age and sex. A similar trend is observed across the models whereby running out of money for food or utilities by the end of the month and lower food security score results in a higher percentage increase in either of total food group, grain, root, and tuber food group consumption, and flesh group consumption.

### 3.6. Infant Food Security Indicators and Meeting the MDD Examined Using Multivariable Logistic Regression Analysis

Multivariable logistic regression results, which adjusted for age and sex, did not find significant associations between meeting the MDD and running out of money for food by the end of the month, being defined as food insecure by the adapted infant food security index and having a low household income (see Table A1).

## 4. Discussion

As hypothesized, the indicator of nutritional risk which had the strongest association with the diet quality of NHPIF infants 3–12 months was the adapted infant food security index, which takes into account multiple indicators of food insecurity. Significant associations after adjusting for infant age and sex were only found with the adapted infant food security index and the two modified USHFSSM questions regarding food group consumption as an indicator of dietary quality. Food-insecure NHPIF infants classified by the adapted infant food security index used in this study consumed a greater number of food groups on average each day, had a greater intake of flesh foods and a greater intake of grains, roots and tubers compared to those classified as food secure by the index. Households who experienced running out of money for food or utilities by the end of the month were significantly associated with greater total food group and more frequent daily flesh food consumption compared to those who did not experience running out of money for food or utilities by the end of the month. However, significant differences in the intake of legumes and nuts, dairy products, eggs, vitamin A-rich fruit or vegetables or other fruit and vegetables were not apparent between infants defined as food secure or insecure by any of the nutritional risk indicators. Based on these findings, the adapted infant food security index may be the better indicator to use to assess the association between food security and food group consumption within this sample of NHPIF infants compared with the two questions modified from the USHFSSM, participation in food assistance programs, annual household income.

Of the infants in this study, 36% met the MDD, on average, each day. This is higher than what has been reported in a cohort of infants aged 8–12 months in Cincinnati, Ohio, where only 28% of infants were found to meet the MDD, on average, each day [25]. In the present study, none of the associations between MDD and any indicators of nutritional risk remained significant in multivariable logistic regression models adjusting for infant age and sex. These findings may be attributed to the small sample size available in this study for infants 6–12 months.

The association between total food group consumption and infant food insecurity by the adapted food security index found in this study was interesting but not unique among the literature. In a study conducted in South Africa, where socioeconomic status (SES) was measured using a composite score of assets and market access, household income, employment status, and educational attainment, MDD was higher among lower SES 6–12-month-old infants. The authors report that their results may have been reflective of the small sample size when stratified by SES and age [23]. A similar justification could be considered for this study whereby infants classified as food insecure by the index used in this study made up only 25.7% (*n* = 18) of the sample. In addition, this study was a secondary analysis and was not sampled to be representative of NHPIF food-insecure infants in Hawai‘i. Thus, the food group consumption identified in this study may not be generalized for the population. These results are suggestive, however, that NHPIF food-insecure infants in Hawai‘i are not at nutritional disadvantage compared to those that are food secure. Rossen L.M et al. similarly reported that food insecurity was largely not associated with dietary intake in a representative sample of 5136 US children aged 2–15 years from NHANES [26]. Likewise, Shinyoung J. et al. did not find a substantial difference in diet quality by household food security or food security among a sample of 5540 children from NHANES 2011–2014 [27]. Other research from a representative sample of New Zealand infants found that food-secure infants had a more diverse diet compared to those who were food insecure [3]. Infants defined as food insecure by the adapted index applied in this study may be employing coping mechanisms such as participation in food assistant programs such as WIC and SNAP. Furthermore, the nutrition of these infants may be prioritized by their caregiver, providing protection against the lack of food resources in the household, as seen in other studies [27].

Similar patterns of higher flesh food intake were identified among lower SES infants in South Africa, as in the results of this study, where the most common type of flesh food consumed daily by low SES infants was processed meat followed by red meat [23]. Processed meats are high in sodium and fat and their intake during infancy has been associated with hypertension and coronary artery disease during adulthood [23,28]. While studies have shown the benefits of flesh foods on infant growth and cognitive development [28,29], the effect of high flesh food consumption in food-insecure infants found in this study was not clear, nor was the type of flesh foods consumed. Meat as a complementary food in infancy is a key source of the micronutrients zinc, iron and vitamin B12 [30]. The pattern of high flesh food intake may contribute to the intake of these micronutrients; however, as this study does not address the type and quantity of foods consumed in each food group, micronutrient intake remains unclear. Factors such as poor maternal nutrition knowledge, delayed introduction of flesh foods, or concerns about potentially allergenic foods [23,31] may influence flesh food consumption patterns during this stage of life.

While authors suggest that dietary diversity is generally associated with child nutritional status and that the associations remain when controlling for household wealth and welfare factors [32], there are drawbacks of assessing infant diet using the global MDD score from the WHO for assessing infant and young child feeding practices. Firstly, food is enumerated when consumed; however, there is no amount recorded. This decision by the creators of the MDD, may be a result of infant portion sizes being small and overall differences in portion sizes having a minimal impact. Secondly, the MDD does not adjust for total energy (kcal). Higher energy intakes could contribute to being overweight during infancy, which is consistently associated with a risk of obesity in childhood and adult life. This association is especially important in populations where the obesity risk is higher such as those of NHPIF ancestry [11]. Thirdly, the designated food groups do not completely distinguish added sugars, sodium and saturated fats. As an example, guidelines for the grains, roots and tubers group does not distinguish a French fry from a boiled wholefood sweet potato. Thus, we identified a greater diversity of foods being eaten by food-insecure infants, however, the quality and quantity of these foods were not assessed using the MDD score. This issue was addressed by Schlichting et al., who added an additional grouping of energy dense nutrient poor foods, which gave an indication of the unhealthy foods consumed [3]. Importantly, the outcome of diet diversity is a concept unique from more traditional dietary quality indices. Furthermore, this dietary assessment method did not incorporate a breastfeeding assessment element. The WHO updated the MDD in 2017 to reflect inclusion of breast milk as the eighth food group [33]. In the present study, breastfeeding status was only considered within the adapted infant food security index.

WIC and SNAP are two important food and nutrition assistance programs conducted by USDA to improve the nutritional well-being of low-income individuals, and there is ongoing interest in investigating the roles of these programs in accomplishing these intentions. One study reported, from a NHANES sample of 1197 children aged 2 to 4 years from low-income households, that WIC food packages are associated with higher diet quality for low-income children [34]. Another study, which addressed the participation and effectiveness of SNAP and WIC in a multi-equation framework for nutrient intakes for young children in the US, found that WIC participation increases the intakes of iron, potassium, and fiber; however, no nutritional effects were found with SNAP participation [35]. The results of this study demonstrate that despite residing in lower income WIC- and SNAP-participating households, these infants may not be at a disadvantage nutritionally compared to those who do not participate in these programs and who are assumed to be of higher income. These results suggest that participation in WIC and SNAP supports more healthful food group consumption. However, further investigation on how these programs mitigate food insecurity and diet quality are needed to inform program implementation in the NHPIF population.

The strengths of this study include the application of an adapted infant food security index which classified food security at the level of the infant and incorporated various indicators of food security. However, this adapted index did not undergo any tests for validity and it did not include the complete 18-item USHFSSM, nor the eight items specific to determining child food security, which would have provided another indicator of food security status. Another strength of the study is that it is the first known examination of food security in this particular population, which acts to fill the relative deficit of such data and provides a base of work for further research. This collection of infant dietary intake by an image-based mFR^TM^ served to reduce the confounding of results, which can occur due to misreporting dietary intake [16]. The mFR^TM^ images enabled a more accurate distinction of foods into appropriate food groups, giving more confidence to the assessment of diet by diversity. Given the cross-sectional nature of this study, only associations can be estimated. In addition, this study was unable to indicate portion size, report on the types of foods consumed within each food group or assess the nutrient quality of foods consumed. This study was only able to report on whether different types of food were consumed. Additionally, the small sample size in this secondary data analysis may have limited the statistical power, and may not be representative of NHPIF infants residing on O‘ahu, Hawai‘i.

## 5. Conclusions

This study investigated various nutritional risk indicators and examined their association with MDD and individual and total food group intake in NHPIF infants. Infants defined as food secure based on the adapted infant food security index had greater overall, flesh food, and grain, tuber and root consumption compared to those defined as food secure. The caregivers of these infants may be employing coping mechanisms such as participation in food assistance programs such as WIC and SNAP. Likewise, these infants may be protected from the effects of food insecurity as their nutrition is prioritized by their caregivers. Of the nutritional risk indicators examined, two questions modified from the USHFSSS, participation in food assistance programs, an adapted infant food security index, and household income, the adapted infant food security index was shown to be the best indicator for consuming more food groups. Further research is needed on a more representative sample of NHPIF infants to determine the most appropriate indicator for food security risk and MDD.

## Figures and Tables

**Table 1:** The adapted infant food security index ^a^ components, weights, scores, and ranges applied to this study.

Adapted Infant Food Security Index Used in this Study				
Component		Weight	Min	Max
Coping	Money for food runs out by the end of the month	Never = 1 Seldom = 0 Sometimes = −1 Most times = −2 Always = −3	−3	1
	Money for utilities runs out by the end of the month	Never = 1 Seldom = 0 Sometimes = −1 Most times = −2 Always = −3	−3	1
	Participation in WIC	Yes = −2 No = 0	−2	0
	Participation in SNAP	Yes = −2 No = 0	−2	0
	Receives reduced cost/free school meals	Yes = −2 No = 0	−2	0
	Receives other food assistance	Yes = −2 No = 0	−2	0
BF	Breastfeeding to 3 months	Exclusive = +2 BF and Formula Feeding = +1 Formula only or BF < 3 month = 0	0	2
Total Score			−14	4
Add constant of 14			0	18

^a^ Adapted from Schlichting D. et al. [3].

**Table 2:** Food security classifications using an adapted food security index among infants aged 3–12 months in the cross-sectional study (*n* = 70) ^a^.

SD Cut Point	Score Range	Definition	Prevalence, *n* (%)
<−2 SD	<7.12	Extremely food insecure	4 (5.7)
−2 SD ≤ • < −1 SD	7.13–10.88	Highly food insecure	5 (7.1)
−1 SD ≤ • < −0.5 SD	10.89–12.76	Moderately food insecure	9 (12.9)
−0.5 SD ≤ • < +0.5 SD	12.77–16.52	Moderately food secure	16 (22.9)
+0.5 SD ≤ • < +1 SD	16.53–18.40	Highly food secure	31 (44.3)
≥+1 SD	≥18.41	Extremely food secure	0 (0)

SD = standard deviation. ^a^ Total number of responses = 65 as there were 5 (7.1%) incomplete responses.

**Table 3:** Characteristics of food-secure and food-insecure infants and their caregivers included in this cross-sectional study examining food security and Native Hawaiian, Pacific Islander, and Filipino (NHPIF) ^a^ infant diet (*n* = 70).

Characteristics		Total Sample	Food- ^b^ Secure Subsample	Food-Insecure Subsample	*p*-Value *^c^*
Age	Months (mean ± SD)	7.4 ± 2.1	7.2 ± 2.1	8 ± 2.2	
	3–6 months, *n* (%)	14 (20.1)	10 (21.4)	4 (27.9)	0.2
	6–12 months, *n* (%)	56 (79.9)	37 (78.7)	14 (72.1)	
Sex	Boy, *n* (%)	38 (54.3)	27 (57.4)	8 (44.4)	0.4
	Girl, *n* (%)	32 (45.7)	20 (42.6)	10 (55.6)	
Marital Status	Married, *n* (%)	46 (61.3)	36 (76.6)	10 (55.6)	0.02
	Single/divorced/widowed, *n* (%)	24 (31.9)	11 (23.4)	8 (44.4)	
Highest Level of Education Attended	College, *n* (%)	50 (66.6)	37 (78.7)	9 (50)	0.03
	Grade School (Elementary–High School), *n* (%)	20 (26.6)	10 (21.3)	9 (50)	
Employed for Wages	Yes, *n* (%)	42 (60)	28 (59.6)	10 (55.6)	0.8
	No, *n* (%)	28 (40)	19 (40.4)	8 (44.4)	
Annual Household Income	>$35,000	48 (81.4)	38 (90.5)	10 (58.8)	0.01
	<$35,000	11 (18.6)	4 (9.5)	7 (41.2)	

^a^ Native Hawaiian, Pacific Islander or Filipino ethnicity. ^b^ As categorized by the adapted infant food security index used in this study. ^c^ *p*-values comparing food-secure and food-insecure subsamples.

**Table 4:** Proportion of infants aged 3–12 months enrolled in the cross-sectional study classified as food secure or insecure by each component of the adapted infant food security index used in this study (*n* = 70) ^a,b^.

Component	Food Security Status				Chi-Squared *p*-Value
Coping			**Food Secure** ***n* (%)**	**Food Insecure** ***n* (%)**	
	Money for food runs out by the end of the month	Yes No	3 (6.4) 44 (93.6)	15 (83.3) 3 (16.7)	0.001
	Money for utilities runs out by the end of the month	Yes No	6 (11.5) 46 (88.5)	12 (92.3) 1 (7.7)	<0.0001
	Participation in Special Supplemental Nutrition Program for Women (WIC)	Yes No	7 (14.9) 40 (85.1)	15 (83.3) 3 (16.7)	<0.0001
	Participation in Supplemental Nutrition Assistance Program (SNAP)	Yes No	3 (6.4) 44 (93.6)	10 (55.6) 8 (44.4)	<0.0001
	Receives reduced cost/free school meals	Yes No	0 (0) 47 (100)	7 (38.9) 11 (61.1)	0.001
	Receives food assistance (Food Bank/Food Pantries or Commodity Foods)	Yes No	0 (0) 47 (100)	2 (11.1) 16 (88.9)	0.07
Breastfeeding	Exclusive breastfeeding to 3 months		30 (63.8)	14 (77.8)	0.7
	Breast and formula feeding to 3 months		13 (28.7)	2 (11.1)	0.2
	Formula only or breastfeeding < 3 months		4 (8.5)	2 (11.1)	0.1

^a^ Adapted from Schlichting D. et al. [3]. ^b^ Total number of responses were 65. 5 (7.1%) incomplete responses.

**Table 5:** Mean percent daily food group consumption by various indicators of food security applied in this analysis for infants aged 3–12 months enrolled in the cross-sectional study (*n* = 70).

	Food Security Indicator																						
	Infants Total (%)	FSSS Q1 ^a^			FSSS Q2 ^b^			WIC			SNAP			Index Score ^c^					Income				
Food Group		Yes (%)	No (%)	*p*	Yes (%)	No (%)	*p*	Yes (%)	No (%)	*p*	Yes (%)	No (%)	*p*	FIS (%)	FS (%)	*p*	Corr	*p*	Low Income (%)	High Income (%)	*p*	Corr	*p*
**Grains, roots and tubers**	85	96	79	0.001	96	82	0.089	93	80	0.023	95	82	0.173 ¥	96	80	0.002	−0.359	0.003	94	82	0.030	−0.227	0.058
**Legumes and nuts**	8	4	9	0.505 ¥	6	8	0.948 ¥	6	9	0.973 ¥	11	7	0.085 ¥	6	9	0.935 ¥	−0.042	0.742	13	7	0.251	−0.132	0.275
**Dairy products**	61	65	60	0.659	48	63	0.283	69	56	0.252	68	59	0.621 ¥	58	62	0.802	−0.016	0.902	77	59	0.224	−0.177	0.143
**Flesh foods**	28	57	17	0.000	63	20	0.000	33	24	0.338	40	25	0.203	61	16	0.000	−0.362	0.003	50	24	0.085	−0.187	0.122
**Eggs**	8	10	7	0.469	6	8	0.767	9	7	0.628	17	6	0.116	12	6	0.146 ¥	−0.002	0.987	20	5	0.067	−0.141	0.247
**Vitamin A-rich fruits and vegetables**	56	65	54	0.192	71	53	0.071	61	54	0.398	63	55	0.570 ¥	63	54	0.361	−0.220	0.078	67	54	0.171	−0.095	0.432
**Other fruits and vegetables**	73	78	71	0.487	85	71	0.186	74	72	0.680 ¥	75	72	0.944 ¥	78	71	0.450	−0.138	0.274	77	72	0.666	−0.056	0.643
**Total food groups (*n*)**	3.2	3.8	3.0	0.005	3.8	3.0	0.018	3.5	3.0	0.110	3.7	3.1	0.055	3.8	3.0	0.008	−0.317	0.010	4.0	3.0	0.003	−0.294	0.014

¥ indicates Mann–Whitney U test result. Special Supplemental Nutrition Program for Women, Infants and Children (WIC), The Supplemental Nutrition Assistance Program (SNAP), *p* value (*p*), correlation (Corr, Spearman’s rho), grains, roots and tubers (Grains), legumes and nuts (Legumes), vitamin A-rich fruits and vegetables (Vitamin A), other fruits and vegetables (Other), ^a^ Adapted USDA Food Security Survey Scale Question. Money for food runs out by the end of the month. No = Never/seldom. Yes = Sometimes/most times/always. ^b^ Adapted USDA Food Security Survey Scale Question. Money for utilities runs out by the end of the month. No = Never/seldom. Yes = Sometimes/most times/always. ^c^ Adapted Infant Food Security Index used in this study. Classified as food secure (FS). Classified as food insecure (FIS).

**Table 6:** Proportion of infants aged 6–12 months enrolled in the cross-sectional study meeting the Minimum Dietary Diversity (MDD) score ^a^ by the various indicators of food security examined in this study (*n* = 70) ^b^.

		Met MDD *n* (%)	Did not Meet MDD *n* (%)	Chi Square *p* Values
**Total**		20 (35.7)	36 (64.3)	-
**Money for food running out by the end of the month ^c^**	No	10 (26.3)	28 (73.7)	0.052
	Yes	8 (57.1)	6 (42.9)	
**Money for utilities running out by the end of the month ^d^**	No	13 (31)	29 (69)	0.173
	Yes	6 (54.5)	5 (45.5)	
**Participation in WIC**	No	10 (28.6)	25 (71.4)	0.165
	Yes	10 (47.6)	11 (52.4)	
**Participation in SNAP**	No	13 (29.5)	31 (70.5)	0.092
	Yes	7 (58.3)	5 (41.7)	
**Food Security Index Status ^e^**	Extremely food insecure	2 (66.7)	1 (33.3)	0.046
	Highly food insecure	2 (50)	2 (50)	
	Moderately food insecure	5 (71.4)	2 (28.6)	
	Moderately food secure	2 (16.7)	10 (83.3)	
	Highly food secure	7 (28)	18 (72)	
	Extremely food secure	0 (0)	0 (0)	
**Food Security Index Classification ^e^**	Food insecure	9 (24.3)	28 (75.7)	0.019
	Food secure	9 (64.3)	5 (35.7)	
**Annual Household Income ($)**	<10,000	4 (80)	1 (20)	0.008
	10,000–20,000	1 (50)	1 (50)	
	20,000–35,000	3 (60)	2 (40)	
	35,000–60,000	3 (42.9)	4 (57.1)	
	60,000–75,000	1 (16.7)	5 (83.3)	
	>75,000	7 (28)	18 (72)	
	<35,000	8 (66.7)	4 (33.3)	0.038
	>35,000	11 (28.9)	27 (71.1)	

Special Supplemental Nutrition Program for Women, Infants and Children (WIC), The Supplemental Nutrition Assistance Program (SNAP). ^a^ Minimum Dietary Diversity Score. ^b^ Total number of responses 65. 5 (7.1%) incomplete responses. ^c^ No = Never/seldom. Yes = Sometimes/most times/always. ^d^ No = Never/seldom. Yes = Sometimes/most times/always. ^e^ Based on the adapted Infant Food Security Index used in this study.

**Table 7:** The association between infant food security indicators and total and individual food group consumption in infants 3–12 months examined using linear regression (*n* = 70) ^a^.

Total Food Group Consumption									
	Model 1			Model 2			Model 3		
Running out of money for food by the end of the month	B	SE	*p*-value	B	SE	*p*-value	B	SE	*p*-value
Constant	2.961	0.149	<0.0001	1.197	0.411	0.01	0.957	0.503	0.1
Running out of money for food by the end of the month	0.831	0.285	0.01	0.728	0.251	0.01	0.689	0.256	0.01
Age				0.242	0.053	<0.0001	0.238	0.054	<0.0001
Sex							0.190	0.229	0.4
R-Squared	0.117			0.333			0.341		
Adjusted R-Squared	0.103			0.312			0.309		
	Model 4			Model 5			Model 6		
Running out of money for utilities by the end of the month	B	SE	*p*-value	B	SE	*p*-value	B	SE	*p*-value
Constant	3.044	0.146	<0.0001	1.235	0.434	0.01	0.795	0.528	0.1
Running out of money for utilities by the end of the month	0.802	0.332	0.02	0.653	0.296	0.03	0.664	0.293	0.03
Age				0.247	0.057	<0.0001	0.240	0.056	<0.0001
Sex							0.333	0.231	0.2
R-Squared	0.082			0.293			0.316		
Adjusted R-Squared	0.068			0.271			0.283		
	Model 7			Model 8			Model 9		
Food Security Score ^b^	B	SE	*p*-value	B	SE	*p*-value	B	SE	*p*-value
Constant	4.307	0.449	<0.0001	2.350	0.586	<0.0001	1.970	0.676	0.01
Food Security Score	−0.279	0.107	0.01	−0.247	0.094	0.01	−0.237	0.094	0.02
Age				0.246	0.055	<0.0001	0.241	0.055	<0.0001
Sex							0.258	0.230	0.3
R-Squared	0.097			0.319			0.333		
Adjusted R-Squared	0.083			0.297			0.300		
**Grain, root and tuber consumption**									
	Model 10			Model 11			Model 12		
Food security score ^b^	B	SE	*p*-value	B	SE	*p*-value	B	SE	*p*-value
Constant	1.117	0.108	<0.0001	0.907	0.158	<0.0001	0.842	0.184	<0.0001
Food Security Score	−0.068	0.026	0.01	−0.064	0.025	0.01	−0.062	0.026	0.03
Age				0.026	0.015	0.1	0.026	0.015	0.1
Sex							0.044	0.063	0.5
R-Squared	0.099			0.143			0.150		
Adjusted R-Squared	0.084			0.115			0.108		
**Flesh food consumption**									
	Model 13			Model 14			Model 15		
Running out of money for food by the end of the month	B	SE	*p*-value	B	SE	*p*-value	B	SE	*p*-value
Constant	0.172	0.050	0.001	−0.413	0.139	0.004	−0.416	0.171	0.02
Running out of money for food by the end of the month	0.398	0.096	<0.0001	0.364	0.085	<0.0001	0.363	0.087	<0.0001
Age				0.080	0.018	<0.0001	0.080	0.018	<0.0001
Sex							0.002	0.078	0.98
R-Squared	0.211			0.398			0.398		
Adjusted R-Squared	0.198			0.379			0.369		
	Model 16			Model 17			Model 18		
Running out of money for utilities by the end of the month	B	SE	*p*-value	B	SE	*p*-value	B	SE	*p*-value
Constant	0.204	0.048	<0.0001	−0.376	0.143	0.01	−0.468	0.176	0.01
Running out of money for utilities by the end of the month	0.431	0.109	<0.0001	0.383	0.098	<0.0001	0.386	0.098	<0.0001
Age				0.079	0.019	<0.0001	0.078	0.019	<0.0001
Sex							0.070	0.077	0.4
R-Squared	0.194			0.370			0.378		
Adjusted R-Squared	0.182			0.351			0.349		
	Model 19			Model 20			Model 21		
Food security score ^a^	B	SE	*p*-value	B	SE	*p*-value	B	SE	*p*-value
Constant	0.753	0.156	<0.0001	0.103	0.206	0.6	0.052	0.240	0.8
Food Security Score	−0.117	0.037	0.003	−0.106	0.033	0.002	−0.105	0.034	0.003
Age				0.082	0.019	<0.0001	0.081	0.019	<0.0001
Sex							0.035	0.082	0.7
R-Squared	0.135			0.330			0.332		
Adjusted R-Squared	0.122			0.309			0.299		

Special Supplemental Nutrition Program for Women, Infants and Children (WIC), The Supplemental Nutrition Assistance Program (SNAP). Model 1 + 13: Running out of money for food by the end of the month. Model 2 + 14: Running out of money for food by the end of the month + age. Model 3 + 15: Running out of money for food by the end of the month + age + sex. Model 4 + 16: Running out of money for utilities by the end of the month. Model 5 + 17: Running out of money for utilities by the end of the month + age. Model 6 + 18: Running out of money for utilities by the end of the month + age + sex. Model 7, 10 + 19: Food Security Score. Model 8, 11 + 20: Food Security Score + age. Model 9, 12 + 21: Food Security Score + age + sex. ^a^ Total number of responses 65. 5 (7.1%) incomplete responses. ^b^ Based on the adapted Infant Food Security Index used in this study.

## Appendix A

**Table A1:** Multinomial logistic regression results examining the association between food security indicators and meeting the MDD in infants 6–12 Months (*n* = 56).

Model 1	B	SE	*p*-Value	OR	95% CI
Intercept	−0.693	0.612	0.258	-	
Household income <$35,000	1.591	0.709	0.025	4.909	1.223–19.709
**Model 2**					
Intercept	7.650	2.543	0.003	-	
Age	−1.006	0.304	0.001	0.366	0.202−0.663
Household income <$35,000	1.736	0.896	0.053	5.672	0.979–32.855
**Model 3**					
Intercept	8.842	2.905	0.002	-	
Age	−0.994	0.305	0.001	0.370	0.204−0.673
Sex	−0.800	0.778	0.304	0.449	0.098–2.065
Household income <$35,000	1.647	0.908	0.070	5.191	0.875–30.779
**Model 4**					
Intercept	−0.288	0.540	0.594	-	
Running out of money for food by the end of the month	1.317	0.654	0.044	3.733	1.037–13.445
**Model 5**					
Intercept	7.751	2.585	0.003	-	
Age	−0.923	0.290	0.001	0.397	0.225−0.701
Running out of money for food by the end of the month	1.037	0.755	0.170	2.820	0.642–12.392
**Model 6**					
Intercept	8.810	2.905	0.002	-	
Age	−0.908	0.289	0.002	0.404	0.229−0.711
Sex	−0.714	0.750	0.341	0.489	0.113–2.127
Running out of money for food by the end of the month	0.842	0.785	0.283	2.321	0.499–10.803
**Model 7**					
Intercept	−0.588	0.558	0.292	-	
Food security index score	1.723	0.677	0.011	5.600	1.487–21.096
**Model 8**					
Intercept	7.081	2.587	0.006	-	
Age	−0.873	0.288	0.002	0.418	0.238−0.734
Food security index score	1.387	0.775	0.073	4.003	0.877–18.271
**Model 9**					
Intercept	8.039	2.898	0.006	-	
Age	−0.862	0.288	0.003	0.422	0.240−0.743
Sex	−0.641	0.757	0.397	0.527	0.120–2.323
Food security index score	1.229	0.799	0.124	3.419	0.715–16.356

Model 1–3: food security indicator: Household income <$35,000. Model 4–6: food security indicator: Running out of money for food by the end of the month. Model 6–8: food security indicator: Food security index score. Model 1, 4, 7: Food security indicator. Model 2, 5, 8: Food security indicator + age. Model 3, 6, 9: Food security indicator + age + sex.
